# Brain Aromatase Modulates Serotonergic Neuron by Regulating Serotonin Levels in Zebrafish Embryos and Larvae

**DOI:** 10.3389/fendo.2018.00230

**Published:** 2018-05-09

**Authors:** Zulvikar Syambani Ulhaq, Mitsuyo Kishida

**Affiliations:** Graduate School of Science and Technology, Kumamoto University, Kumamoto, Japan

**Keywords:** estradiol, brain aromatase, biphasic manner, serotonergic neuron, zebrafish, early development

## Abstract

Teleost fish are known to express two isoforms of P450 aromatase, a key enzyme for estrogen synthesis. One of the isoforms, brain aromatase (AroB), *cyp19a1b*, is highly expressed during early development of zebrafish, thereby suggesting its role in brain development. On the other hand, early development of serotonergic neuron, one of the major monoamine neurons, is considered to play an important role in neurogenesis. Therefore, in this study, we investigated the role of AroB in development of serotonergic neuron by testing the effects of (1) estradiol (E_2_) exposure and (2) morpholino (MO)-mediated AroB knockdown. When embryos were exposed to E_2_, the effects were biphasic. The low dose of E_2_ (0.005 µM) significantly increased serotonin (5-HT) positive area at 48 hour post-fertilization (hpf) detected by immunohistochemistry and relative mRNA levels of tryptophan hydroxylase isoforms (*tph1a, tph1b*, and *tph2*) at 96 hpf measured by semi-quantitative PCR. To test the effects on serotonin transmission, heart rate and thigmotaxis, an indicator of anxiety, were analyzed. The low dose also significantly increased heart rate at 48 hpf and decreased thigmotaxis. The high dose of E_2_ (1 µM) exhibited opposite effects in all parameters. The effects of both low and high doses were reversed by addition of estrogen receptor (ER) blocker, ICI 182,780, thereby suggesting that the effects were mediated through ER. When AroB MO was injected to fertilized eggs, 5-HT-positive area was significantly decreased, while the significant decrease in relative *tph* mRNA levels was found only with *tph2* but not with two other isoforms. AroB MO also decreased heart rate and increased thigmotaxis. All the effects were rescued by co-injection with AroB mRNA and by exposure to E_2_. Taken together, this study demonstrates the role of brain aromatase in development of serotonergic neuron in zebrafish embryos and larvae, implying that brain-formed estrogen is an important factor to sustain early development of serotonergic neuron.

## Introduction

Biosynthesis of estrogen is catalyzed by the action of cytochrome P450 aromatase, a product of *cyp19a1* gene (1, 2). Contrary to mammals, zebrafish and many other teleosts have two isoforms of aromatase gene, *cyp19a1a* and *cyp19a1b*, encoding ovarian and brain aromatase, respectively (3, 4), and their predominant expression in respective tissues indicates differential regulation and functions. Fish brain is characterized by having much higher aromatase expression in brain compared to mammals (5). At the same time, fish brain has been reported to exhibit elevated neuroregenerative capacity compared to mammals (6–8). Widespread proliferation zones are detected in zebrafish brain (6, 9), while only limited areas such as subependymal and subgranular zones exhibit proliferation in mammals (7). Such high neurogenic activity in teleost fish may be attributed to increased synthesis of estrogen due to the elevated expression of brain aromatase. Indeed, expression of brain aromatase is localized in radial glial cells (RGCs), which differentiate into neurons and other glial cells contributing to adult neurogenesis as well as developmental neurogenesis (10–12). Developmental studies in zebrafish show that expression of brain aromatase in embryos increases rapidly after 12 hour post-fertilization (hpf), and is regulated by positive feedback loop through its own product, estrogen, acting on estrogen response element of *cyp19a1b* (3, 13, 14). Therefore, the zebrafish model expressing elevated levels of brain aromatase in early development is suitable to investigate the functional significance of aromatase and neural estrogen in developing brain.

Serotonin (5-HT), a neurotransmitter produced by multiple enzymatic steps including a rate-limiting action of tryptophan hydroxylase (TPH), plays a major role in a number of physiological processes and pathological conditions, such as depression (15, 16), stress (15, 17), cardiac function (18), reward seeking behavior (19), and anxiety (15, 20). In addition, serotonergic neuron is known to be involved in neurogenic activities (21). It has been reported that 5-HT is critically involved in the brain plasticity, neural trafficking, synapse formation, and network construction during development (22, 23). Serotonergic neurons in raphe nuclei extend their axons to the forebrain possibly modulating the differentiation of neuronal progenitors (24). Early ontogeny of serotonergic system may further suggest its role in brain development (25). Raphe 5-HT populations in human brain are considered as the earliest to be identified (24, 26).

Serotonergic neurons in mammalian brain are localized mainly in raphe nuclei of brain stem, which project into accumbens, hypothalamus, substantia nigra, and periaqueductal gray (22, 23). On the other hand, 5-HT-positive cell bodies are detected mainly in three populations in adult fish brain: pretectal area, posterior tuberculum/hypothalamus, and raphe (27, 28). Interestingly, distributions of serotonergic populations and their fibers overlap with highly proliferative areas of fish brain, which may indicate serotonergic regulation in adult neurogenesis in fish (27). In adult zebrafish, serotonin has been shown to promote regeneration of motor neurons by acting on progenitor cells (29).

It is well documented that serotonergic neuron is one of the targets of estrogen in mammals (30, 31). In macaques, estrogen increases gene expression and protein contents for TPH (32), and decreases gene expression of the serotonin reuptake transporter and the 5HT1A autoreceptor (33, 34). In mammals, both ERα and ERβ are expressed in 5-HT neurons with differential distributions depending on species and sex (35–37). ERβ has been shown to regulate *tph2* expression in serotonergic neurons (38, 39). Similarly in teleost fish, effects of ovarian steroids on serotonin system have been reported in some species. In tilapia, the response of 5-HT content in brain to E_2_ treatment was dependent on developmental stages. Treatment between days 7 and 10 posthatching decreased 5-HT content, while the treatment at later stages increased it (40). Similar result was obtained in Japanese sea bass, which shows a significant decrease in brain 5-HT content in fingerlings after E_2_ treatment, while the content increased in juvenile group (41). Indeed, overlapping distributions of ER with raphe 5-HT innervation in telencephalon and diencephalon of adult zebrafish brain implies close association of ER and serotonergic neurons (27, 42). It has been reported that ERβ exhibits broad distribution along the brain ventricles of telencephalon and diencephalon in adult zebrafish (43), though co-localization of ER in serotonergic neurons has yet to be documented in fish.

Therefore, in this study, we tested the hypothesis that brain aromatase modulates serotonergic neuron in early development of zebrafish. In order to elucidate a possible role of brain-formed estrogen, we first examined the effects of exogenous E_2_ and then MO-mediated knockdown of brain aromatase on parameters such as 5-HT contents, relative *tph* expression levels, heart rate, and thigmotaxis in zebrafish embryos and larvae.

## Materials and Methods

### Fish Maintenance and Embryo Culture

Adult zebrafish (*Danio rerio*) were obtained from the local pet shop and reared in a 60-L tank. Water temperature was maintained at 26–30°C, and the light regime was 14 h of light starting at 10:00 followed by 10 h of dark. Fish were fed with TetraMin (Tetra Japan Inc.) twice a day. Fertilized eggs were collected within 15 min after fertilization and washed in embryo medium (EM) (0.004% CaCl_2_, 0.163% MgSO_4_, 0.1% NaCl, and 0.003% KCl) to remove debris. Embryos were transferred to a 6-well plastic plate (30 embryos in 8 mL of EM per well), and incubated at 28 ± 0.5°C. The medium was changed daily. All experimental procedures and maintenance of fish were conducted in accordance with the Guide for Care and Use of Laboratory Animals published by the US National Institutes of Health.

### Exposure Experiments

Stock solutions of 17β-estradiol (E_2_) (Sigma-Aldrich) at 10 mM, ICI 182,780 (ICI) (Tocris Bioscience) at 10 mM, and dexamethasone (DEX) (Wako) at 100 mM were prepared in dimethyl sulfoxide (DMSO), and diluted with EM to the final concentrations indicated in the experiments. Quipazine maleate salt (Q) (Sigma-Aldrich) and fluoxetine hydrochloride (FLX) (Wako) were dissolved in ethanol at 100 and 10 mM, respectively, which were further diluted with EM to the final concentrations used in the experiments. Control embryos were cultured in 0.1% DMSO or ethanol. Exposure started at 2 hpf and continued till embryos and larvae were subjected to the assays. The media were changed daily.

### Morpholino (MO) Microinjection

Morpholino antisense oligos were purchased from Gene Tool. MO sequences are shown in Table 1. MOs were dissolved in distilled water to 50 mg/mL and stored at −20°C. Before injection, MO solution was heated at 65°C for 5 min and further diluted to the working concentrations (2.5 and 5 ng/nL) with deionized H_2_O containing rhodamine B (Wako). The final concentration of rhodamine B was 0.08%. MO was injected into embryos at one to four cell stages using a glass microcapillary injection needle attached to the automatic nanoliter injector (Drummond Scientific). Injection volume was set at 2.3 nL per embryo. After the injection, embryos were observed under the fluorescence microscope (Leica M165 FC), and embryos that did not exhibit red fluorescence were discarded. To examine the effect of MO-mediated AroB knockdown, AroB MO designed to block translation was injected (2.5 and 5 ng/nL). Uninjected embryos (C), embryos injected with 5 ng/nL of standard control MO (Std MO), and inverted AroB MO (InvB MO) served as control groups. MO to block translation of *cyp19a1a*, ovarian aromatase (AroA MO) was also tested for 5-HT immunohistochemistry. As it has been reported that MO injection will cause off-target effect such as apoptosis through activation of *p53* gene (44, 45), MO to block translation of p53 (p53 MO) at 2 ng/nL was co-injected with AroB MO at 5 ng/nL.

**Table 1:** Morpholino (MO) sequences.

Name	Sequence	Reference
AroA MO (ovarian aromatase MO)	GGAGCAGATCACCTGCCATAAGAAC	This paper
Genebank Acc. No.: AF226620		

InvA MO (inverted ovarian aromatase MO)	CAAGAATACCGTCCACTAGACGAGG	This paper

AroB MO (brain aromatase MO)	ATCCTTTACCACATGCTCCATCATC	This paper
Genebank Acc. No.: AF226619		

InvB MO (inverted brain aromatase MO)	CTACTACCTCGTACACCATTTCCTA	This paper

Std MO (standard control MO)	CCTCTTACCTCAGTTACAATTTATA	Gene tools

p53 MO	GCGCCATTGCTTTGCAAGAATTG	(44, 45)
Genebank Acc. No.: NM 131327		

For rescue experiments, the AroB mRNA (30 pg/nL) was co-injected with AroB MO (5 ng/nL). The full length AroB cDNA was obtained by One Step PrimeScript RT-PCR Kit (Takara) using total RNA from 7-dpf zebrafish larvae and AroB primers (Table 2). Amplified products were purified with NucleoSpin Gel and PCR Clean-up (Machery-Nagel) and subcloned into pGEM-T Easy Vector (Promega). Nucleotide sequences and orientation of the inserts were verified by DNA sequencing analysis carried out using BigDye Terminator v3.1 Cycle Sequencing Kit (Applied Biosystems) and ABI 3130 xl genetic analyzer (Applied Biosystems). Plasmid DNA was linearized with *SalI* and the full length AroB mRNA was transcribed *in vitro* by MAXIscript T7 Kit (Ambion).

**Table 2:** PCR primers and conditions.

Gene	Primer sequence (5′ → 3′)	Size of PCR product (bp)	Amplification profile
*tph1a*	F: TTCAAGGACAATGTCTATCG	214	94°C—30 s
	R: GGGAGTCGCAGTGTTTGATG		55°C—30 s
	Genebank Acc. No.: AF548566 (46)		72°C—60 s (35 cycles)

*tph1b*	F: TACCTGCAGAACCTGCCTCT	430	94°C—30 s
	R: AGAGAAGACCAGCCCCGTAT		55°C—30 s
	Genebank Acc. No.: BC154120 (46)		72°C—60 s (35 cycles)

*tph2*	F: GTGTGAACTCCAAAGCAGCA	684	94°C—30 s
	R: TGGTATTCCTTCCCCATCTG		55°C—30 s
	Genebank Acc. No.: AB125219 (46)		72°C—60 s (35 cycles)

*cyp19a1b* (AroB)	F: TTAAAGAGGTGTGTCTGTATGTGAGGTG	1,435	42°C—10 min
	R: GGAATTTACTCTGTGCGCCTTTAAATGT		94°C—30 s
	Genebank Acc. No: BC076104		60°C—15 s
			72°C—90 s (40 cycles)

*β-Actin*	F: GGTATGGGACAGAAAGACAG	330	94°C—30 s
	R: AGAGTCCATCACGATACCAG		58°C—30 s
	Genebank Acc. No: AF025305 (3)		72°C—60 s (34 cycles)

### Western Blot and Dot Blot Analysis

The antiserum to brain aromatase was produced in a rabbit against the synthetic peptide, CNSNGETADNRTSKE of zebrafish AroB (Sigma-Genosys). This peptide sequence has been used to raise the specific antibody as previously described (47). To confirm the specificity of the antiserum, Western blot of brain extract was conducted. Adult female zebrafish were exposed to E_2_ (5 and 25 ng/L) or vehicle alone (0.00025% DMSO) for 24 h (three fish per group). Brains were pooled and homogenized in HBST buffer (100 mM NaCl, 10 mM HEPES, 0.5% Triton-X 100, 0.01% TPCK, and 0.01% TLCK). After centrifugation at 10,000 *g* for 10 min, protein concentrations in supernatant were measured using BCA protein assay kit (Thermo Scientific). Extracts (30 µg protein/sample) were separated on 12.5% SDS-PAGE and transferred to a PVDF membrane. Precision Plus Protein Unstained Standards (Bio-Rad) were used for size reference. After blocking by 1% skim milk in PBS for 1 h, the membrane was incubated with the AroB antiserum (1:500) for 2 h, and then with the secondary antibody conjugated with alkaline phosphatase (AP) (Abcam) (1:1,000) for 1 h. After washing, the membrane was incubated in AP buffer (0.1 M Tris–HCl, pH 9.5, 0.1 M NaCl, 1 M MgCl) for 1 min. Signals were developed for 2–3 min in BCIP/NBT substrate (Roche) diluted at 1:50 in AP buffer, and the reaction was stopped by 0.5 M EDTA. All the incubation steps were done at RT. The antiserum to ovarian aromatase was raised in a rabbit using a synthetic peptide, CKPDVYFRLDWLHKKHKRD of zebrafish AroA (Sigma-Genosys). Similarly, Western blot with the antiserum (1:500) was performed using the ovarian extract prepared with HBST buffer from three adult fish (30 μg/lane).

To examine the effect of MO-mediated AroB knockdown, dot blot analysis using 120 larvae at 6 dpf collected from 4 separate MO injection experiments were pooled and extracted similarly as described for the brain extract. Extracts containing 40 µg protein (3 µL) were spotted onto nitrocellulose membrane (GVS Life Science). The membrane was treated similarly as in Western blot except for the concentration of the secondary antibody at 1:2,000. Density of the blots were analyzed with NIH ImageJ software. Blots of embryo extracts treated with the pre-immunized rabbit serum were used as a negative control to subtract from the density obtained with the AroB antiserum. No changes were observed among controls (uninjected, standard control MO, and inverted AroB MO) (data not shown). The effect of MO-mediated AroA knockdown was also examined by dot blot analysis. Briefly, pooled 120 embryos at 2 dpf collected from 5 separate embryo cohorts were extracted. Extracts containing 30 µg protein (3 µL) were spotted onto nitrocellulose membrane and subjected to immunostaining using AroA antiserum at 1:500. No changes were observed among controls including inverted AroA MO (data not shown).

### 5-HT Immunohistochemistry

Whole-mount immunohistochemistry for 5-HT was carried out according to the previous studies (48, 49). 2-dpf embryos were fixed in 4% paraformaldehyde in PBS overnight at 4°C. Fixed embryos were rinsed in PBS, bleached in 3% H_2_O_2_ for 30 min and stored in methanol at −20°C until use. For immunostaining, embryos were washed in PBS containing 0.1% Tween-20 and 0.5% Triton X-100 (PBSTX), and then permeabilization was achieved by incubation in deionized H_2_O for 60 min at RT followed by 100% acetone for 8 min at −20°C. Non-specific binding was blocked by incubation in 10% normal goat serum (NGS) and 3% BSA for 3 h at RT. After several washes with PBSTX, embryos were incubated in rabbit polyclonal anti 5-HT (ImmunoStar) diluted at 1:500 in 10% NGS/PBS containing 0.3% Triton-X 100 for 2 days at 4°C. After rinsing in PBSTX for 4 h, embryos were incubated in the goat anti-rabbit IgG Alexa Fluor 488 (Molecular Probes Invitrogen Detection Technologies) diluted at 1:100 in 10% NGS/PBS overnight at 4°C. After thorough washing in PBSTX, embryos were mounted in 0.5% agarose and observed under the fluorescence microscope (Leica M165 FC). Negative controls processed by omitting incubation with the primary antibody or by replacing the primary antibody with normal rabbit serum showed no positive signals. For measurement of 5-HT-positive area, focus was adjusted on the field with the largest positive area, and NIH ImageJ software was used to quantify manually outlined areas. Immunostaining was performed using five to eight embryos per group, and the experiments were done in triplicate.

### RT-PCR

Total RNA was extracted from larvae at 4 and 7 dpf (25 larvae/group) using ISOGEN II (Nippon Gene) and treated with DNase free (Ambion). cDNA was synthesized from 1 µg total RNA using Reverse Transcription System (Promega). A total reaction volume of 25 µL containing 2× GoTaq Green Master Mix (Promega), 10 µM of each primer, and 1 µL cDNA was subjected to PCR using Program Temp Control System PC708 (Astec). β-Actin was used as an internal control. Amplification conditions and primer sequences are listed in Table 2. The amplified products were separated on a 2% agarose gel. Levels of mRNAs expression were analyzed by NIH ImageJ software and normalized by the expression level in the control group at each developmental time. Experiments were done in triplicate.

### Heart Rate Measurement

Embryos at 2 dpf were individually placed in a well of a 12-well culture plate containing 500 µL of corresponding experimental medium and kept for 15 min to allow heartbeats to resume a steady rate. Heart beats were counted manually for 15 s under a stereo microscope (Leica 58APO). Ten embryos were used for each group. Experiments were repeated three times with eggs collected from different spawns.

### Thigmotaxis Assay

Assay was performed according to the protocols described previously (49, 50). Briefly, zebrafish larvae at 6 dpf were transferred into a 6-well tissue culture plate with one fish per well containing 4 mL EM. The bottom of each well was divided into two portions designated as inner and outer zones. After habituation at 28°C for 2 h followed by acclimation under the video camera for 5 min, swimming activity was recorded for 5 min. For each group, 12 larvae were used. Data from 36 larvae from three different spawns were pooled and analyzed to express % of time a fish spent in the outer zone.

### Statistical Analysis

Data are presented as mean ± SEM. Statistical differences between groups were evaluated by one-way ANOVA followed by Tukey’s or least significant difference *post hoc* test using IBM SPSS statistics version 19. Unpaired Student’s *t*-test was used for the dot blot analysis. Kruskal–Wallis test and Mann–Whitney *U* test were used for thigmotaxis assays, as the data did not meet the assumptions required for parametric testing. Significant differences were accepted when *p* < 0.05.

## Results

### Effect of E_2_ Exposure on Serotonergic Neuron

**Figure 1:**
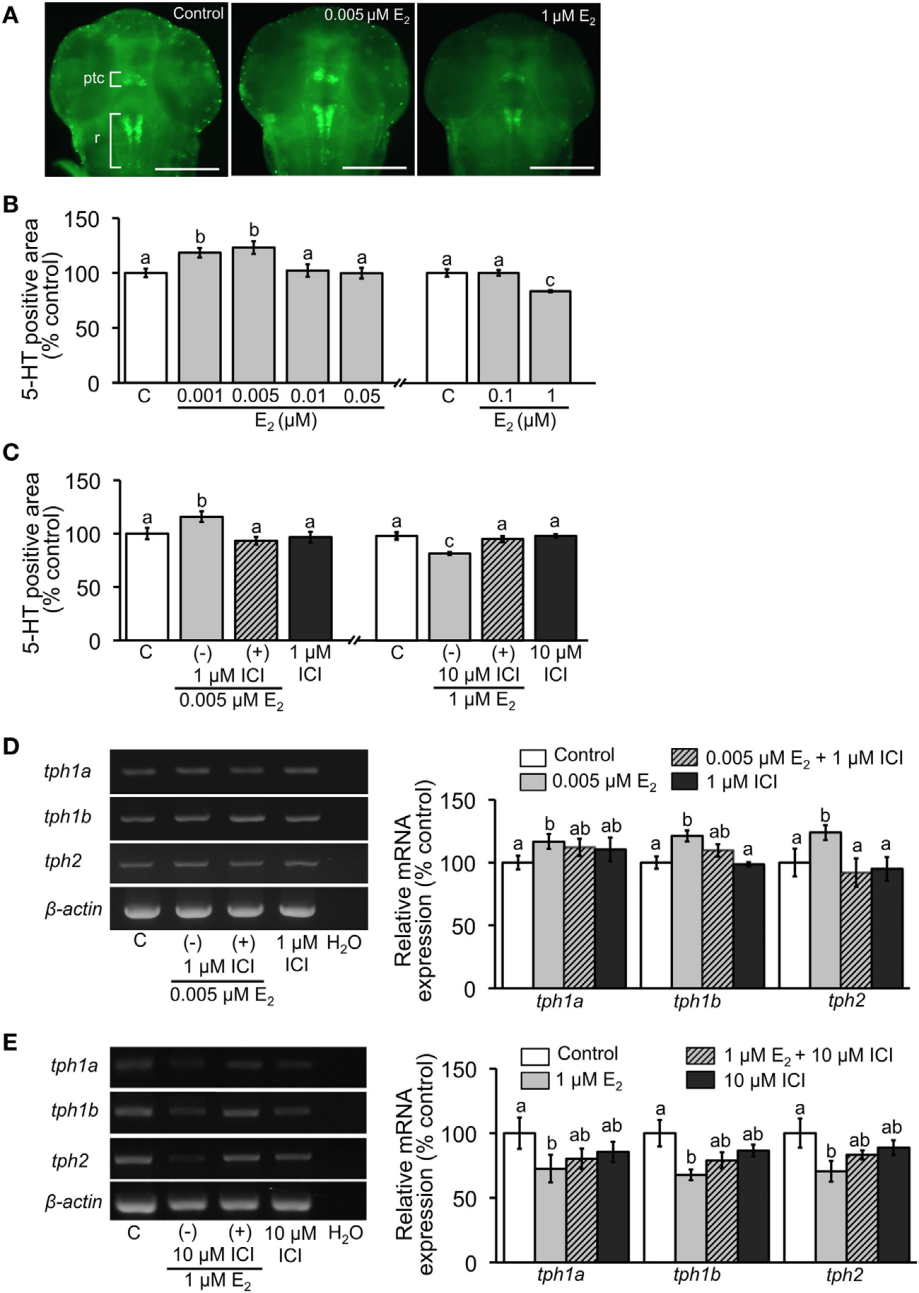
Effect of E_2_ on 5-HT-positive area and relative expression of *tph* isoforms. **(A)** Representative images of ventral view of head region showing 5-HT-positive cells in pretectal and thalamic complex (ptc) and raphe (r) at 2 dpf. Scale bar: 200 µm. **(B,C)** Measurements of the area of 5-HT-positive neurons in the experiment of E_2_ exposure and co-incubation of E_2_ and ICI, respectively. **(D,E)** Semi-quantitative PCR for expression of *tph* isoforms in 4 dpf larvae in the experiments of exposure to low and high doses of E_2_ and co-incubation with ICI, respectively. Data are presented as a mean ± SEM. Different letters in each graph indicate significant differences (*p* < 0.05).

5-HT-positive neurons were detected in the embryos at 2 dpf by whole-mount fluorescent immunohistochemistry (Figure 1A). Positively stained neurons were located in pretectal and thalamic complex and raphe as reported previously (48, 51–54). 5-HT-positive areas were significantly increased when exposed to low doses E_2_ (0.001 and 0.005 µM) but decreased in high dose (1 µM) exposure (Figure 1B). Effects of both low and high doses E_2_ were significantly reversed by addition of 1 or 10 µM ICI, respectively (Figure 1C).

Relative expression levels of *tph* isoforms at 4 dpf were analyzed by semi-quantitative PCR. Significant increase in expression was detected in all *tph* isoforms when embryos were exposed to low-dose E_2_ (0.005 µM). Addition of 1 µM ICI completely reversed the decreased expression of *tph2*, while expressions of *tph1a* and *tph1b* were reversed partially (Figure 1D). Conversely, high-dose E_2_ exposure significantly decreased expression levels of all isoforms, which was partially reversed by addition of 10 μM ICI (Figure 1E).

**Figure 2:**
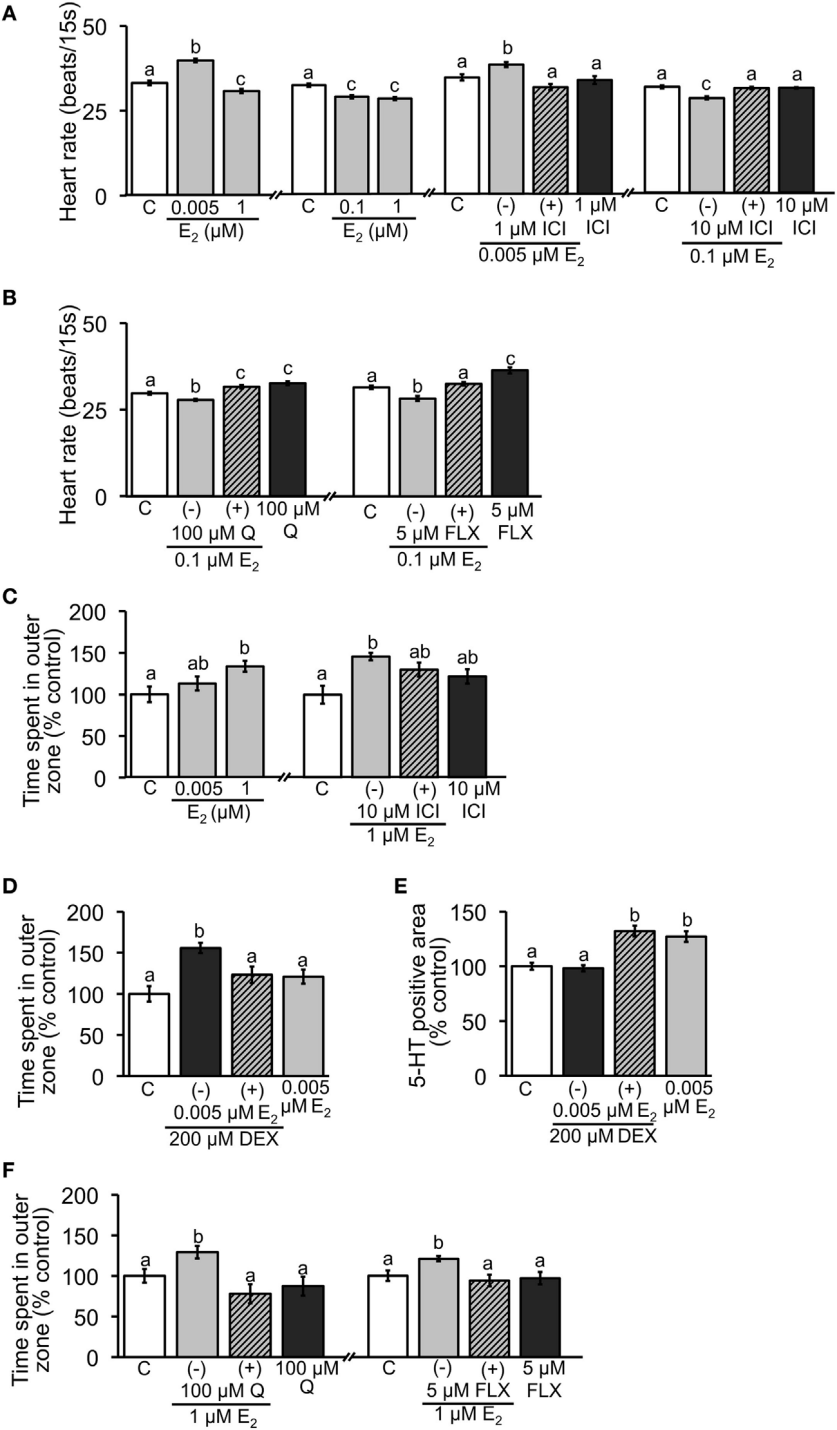
Effect of E_2_ on heart rate and thigmotaxis. Heart rate was measured in 2-dpf embryos; **(A)** exposure to low and high doses of E_2_ and co-incubation with ICI; **(B)** co-incubation of E_2_ and Q or FLX to examine involvement of serotonin signaling. Thigmotaxis assay was performed to evaluate anxiety level in 6-dpf larvae; **(C)** exposure to low and high doses of E_2_ and co-incubation with ICI; **(D)** anxiolytic effect of low dose of E_2_ when larvae were exposed to 200 µM DEX. **(E)** Measurements of 5-HT-positive neuron area in 2-dpf embryos exposed to DEX and low dose of E_2_. **(F)** Effects of co-incubation of E_2_ and Q or FLX on thigmotaxis to examine involvement of serotonin signaling. Data are presented as a mean ± SEM. Different letters in each graph indicate significant differences (*p* < 0.05).

When the embryos were exposed to E_2_, the heart rate at 2 dpf was significantly increased in the 0.005 µM group, while significant decrease was found in the 0.1 and 1 µM groups (Figure 2A). Addition of 1 and 10 µM ICI significantly reversed the effects caused by low or high dose of E_2_, respectively (Figure 2A). To verify the role of serotonergic signaling in regulation of heart rate, effects of Q (5-HT agonist) and FLX (5-HT selective re-uptake inhibitor) were tested. Co-incubation with 0.1 µM E_2_ significantly reversed the decreased heart rate caused by E_2_. Heart rate was significantly increased when exposed to Q or FLX alone (Figure 2B).

Thigmotaxis assay was performed using 6-dpf larvae. Exposure to 1 µM E_2_ increased the time fish spent in outer zone, suggesting that anxiety was increased, but no significant difference was observed in 0.005 µM E_2_ group (Figure 2C). Addition of 10 µM ICI partially reversed the increase caused by 1 µM E_2_ (Figure 2C). To further examine the effect of low-dose E_2_, embryos were exposed to 200 µM DEX and subjected to the assay. DEX alone increased the time, but co-incubation with 0.005 µM E_2_ significantly reduced the increase (Figure 2D). Immunostaining for 5-HT showed that co-incubation of low-dose E_2_ and DEX increased the positive staining, although DEX alone had no effect (Figure 2E). To verify the role of serotonergic signaling in thigmotaxis assay, effects of Q and FLX were tested. Both Q and FLX significantly decreased the time fish spent in outer zone caused by 1 µM E_2_ exposure (Figure 2F).

### Validation of MO-Mediated Knockdown Using the Specific Antisera

**Figure 3:**
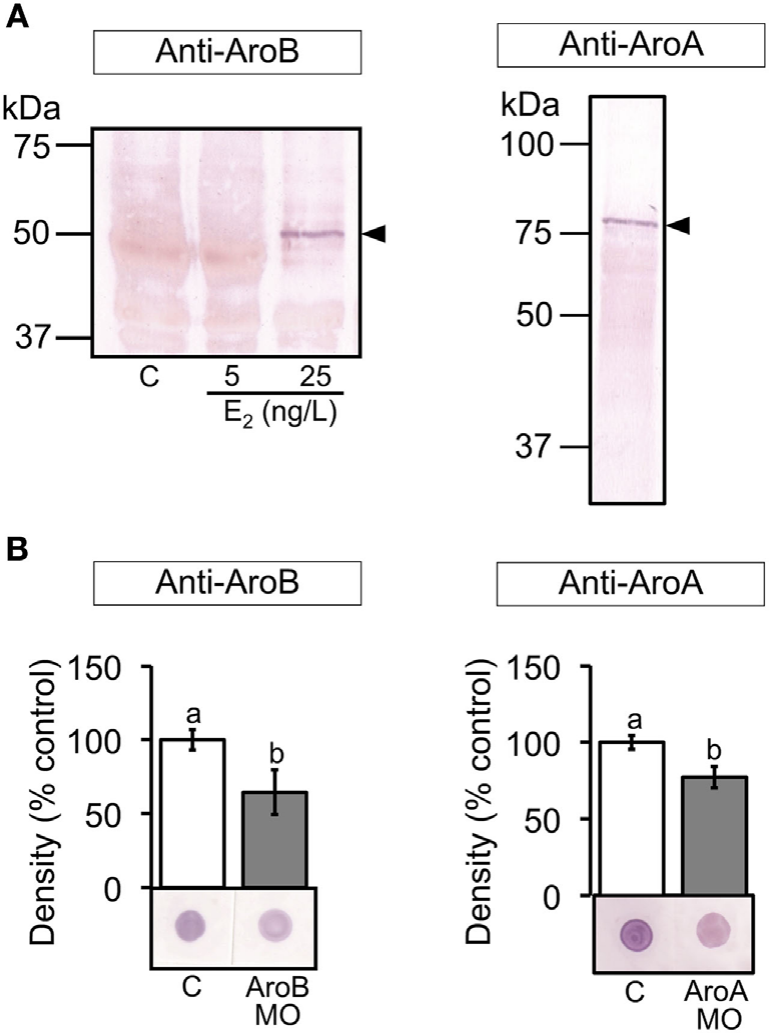
Validation of MO-mediated knockdown on translation. **(A)** Western blots of brain extracts from control and E_2_ exposed fish and ovarian extract from untreated fish were stained with the antiserum to AroB and AroA, respectively, showing a positive band at the expected size for each aromatase as indicated by arrowheads. **(B)** Dot blots of 6 and 2 dpf larval extracts using the antiserum to AroB and AroA, respectively, were analyzed. Representative blots are shown in each graph. Data are presented as a mean ± SEM. Different letters in each graph indicate significant differences (*p* < 0.05).

Specificity of the antisera to AroB and AroA was examined by Western blot. The anti-AroB revealed a single band at the expected size of 50 kDa in brain extract from the fish exposed to E_2_ at 25 ng/L (47) (Figure 3A). The anti-AroA detected a single band at the expected size of 75 kDa in the ovarian extract (Figure 3A), which is in agreement with the previous study (55). In addition, immunohistochemistry of the ovary showed similar localization of AroA as previously described (56) (data not shown).

The dot blot analysis of the larval extracts showed that both AroB and AroA MO injections significantly decreased immunoreactivity compared to the uninjected control, indicating decreased translation of AroB and AroA, respectively (Figure 3B). Std MO, InvB, or InvA MO did not show any significant difference compared to the uninjected control (data not shown).

### Effect of MO-Mediated Knockdown of AroB on Serotonergic Neuron

**Figure 4:**
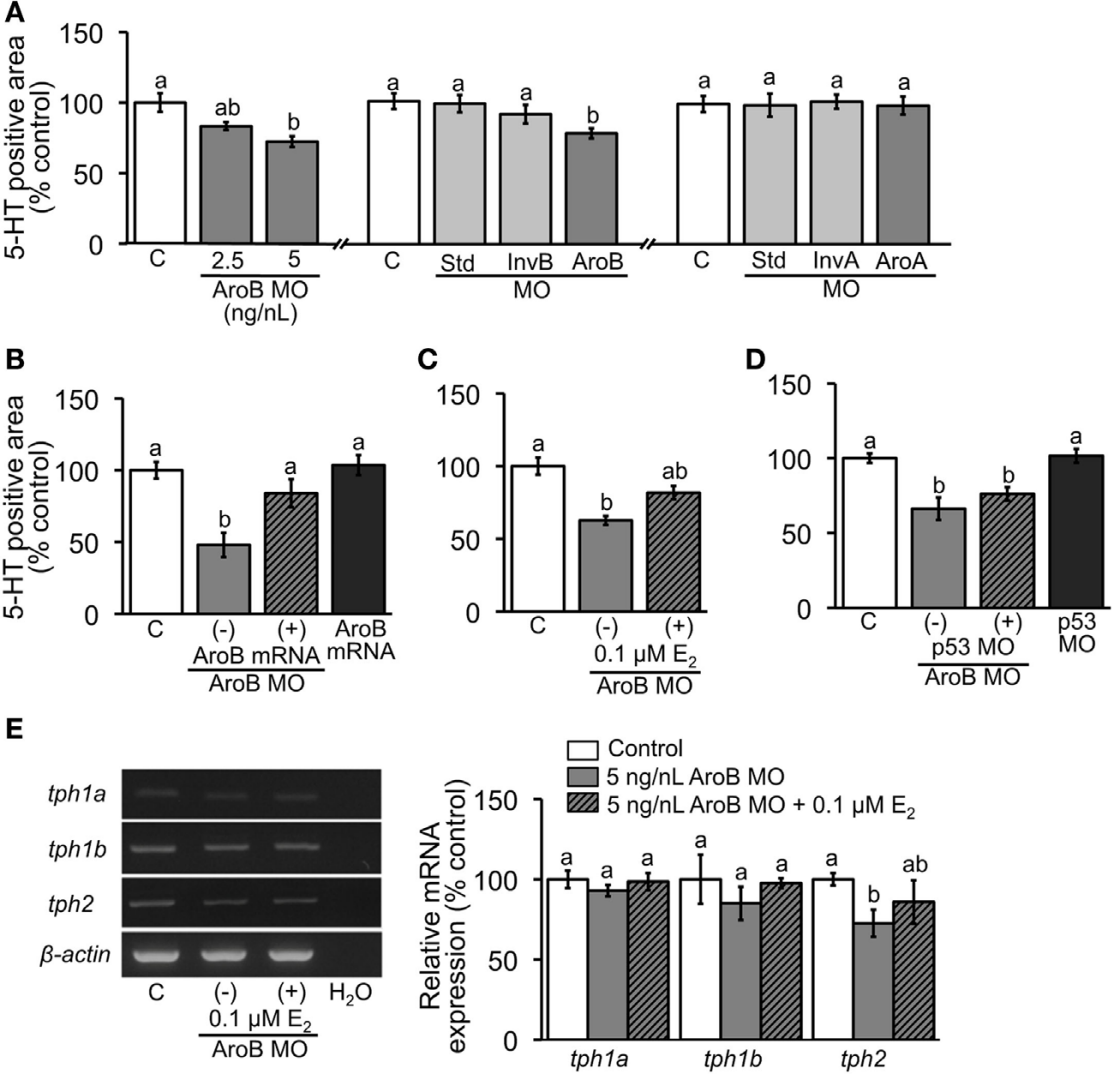
AroB MO-mediated effect on serotonergic neuron. 5-HT-positive neuron area was measured in 2-dpf embryos; **(A)** injection of AroB MO and control MOs (Std MO, InvB MO and AroA MO); **(B,C)** co-injection of AroB mRNA and exposure to E_2_, respectively, to rescue the effect of AroB MO; **(D)** co-injection of p53 MO to test off-target effect of AroB MO. **(E)** Semi-quantitative PCR measurement of expression of *tph* isoforms in 6-dpf larvae injected with AroB MO with and without exposure to E_2_. Data are presented as a mean ± SEM. Different letters in each graph indicate significant differences (*p* < 0.05).

When AroB MO was injected, 5-HT-positive area was significantly decreased in the 5 ng/L group and partially decreased in the 2.5 ng/nL group compared to the uninjected control (Figure 4A). Injections of Std MO and InvB MO did not show any significant difference in 5-HT-positive areas compared to the uninjected control (Figure 4A). Moreover, the injection of AroA did not show any changes (Figure 4A). The decrease in 5-HT-positive area caused by AroB was completely rescued by co-injection of 30 pg/nL AroB mRNA (Figure 4B) and partially rescued by E_2_ exposure at 0.1 µM (Figure 4C). When p53 MO was co-injected with AroB MO to examine off-target effect, no significant difference in 5-HT-positive area was observed, suggesting that decrease in 5-HT-positive area caused by AroB MO is not due to apoptosis caused by *p53* activation (Figure 4D).

The effect of AroB MO injection on relative expression of *tph* isoforms was evaluated by semi-quantitative PCR using 7-dpf larvae. While expression levels of *tph1a* and *tph1b* showed no significant changes, expression of *tph2* isoform was significantly decreased and partially rescued by E_2_ exposure at 0.1 µM (Figure 4E).

**Figure 5:**
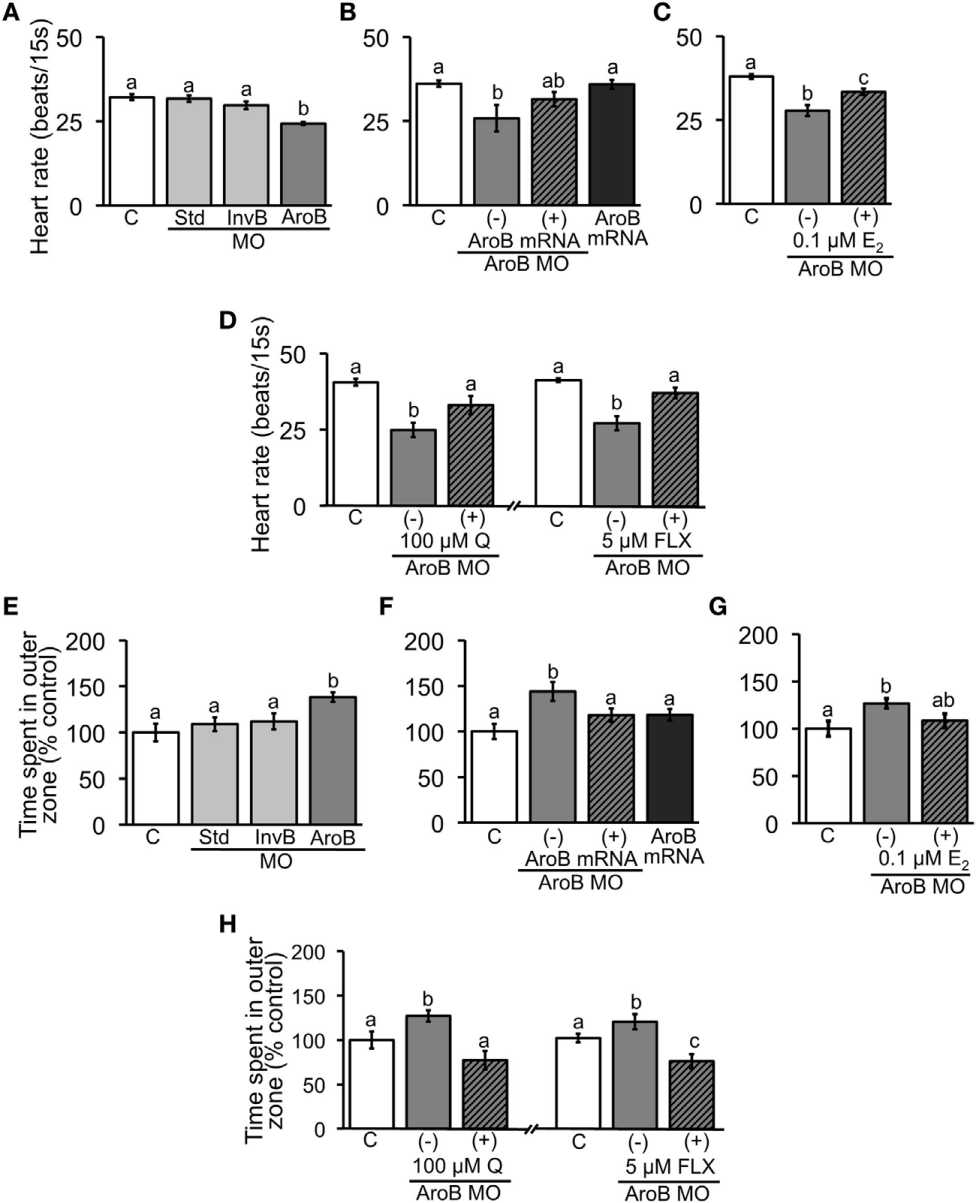
AroB MO-mediated effect on heart rate and thigmotaxis. Heart rate was measured in 2-dpf embryos; **(A)** injection of AroB MO and control MOs; **(B,C)** co-injection of AroB mRNA and exposure to E_2_, respectively, to rescue the effect of AroB MO; **(D)** injection of AroB MO with and without exposure to Q and FLX to examine involvement of serotonin signaling. Thigmotaxis assay was conducted using 6-dpf larvae; **(E)** injection of AroB MO and control MOs; **(F,G)** co-injection of AroB mRNA and exposure to E_2_, respectively, to rescue the effect of AroB MO; **(H)** injection of AroB MO with and without exposure to Q and FLX to examine involvement of serotonin signaling. Data are presented as a mean ± SEM. Different letters in each graph indicate significant differences (*p* < 0.05).

Heart rate of AroB MO injected embryos was significantly decreased compared to the uninjected or Std MO and InvB MO injected controls (Figure 5A). The decrease caused by AroB MO was rescued either by co-injection of AroB mRNA or by exposure to 0.1 µM E_2_ (Figures 5B,C). Exposure to 100 µM Q as well as to 5 µM FLX (Figure 5D) reversed the decrease to the control level.

In thigmotaxis assay, AroB MO injection caused significant increase in time fish spent in outer zone compared to the uninjected, Std MO injected, and InvB MO injected controls (Figure 5E). This effect of AroB MO was rescued either by co-injection of AroB mRNA or by exposure to 0.1 µM E_2_ (Figures 5F,G). Exposure to 100 µM Q as well as to 5 µM FLX reversed the time fish spent in outer zone increased by AroB MO injection (Figure 5H).

## Discussion

The aim of this study is to elucidate the role of estradiol and brain aromatase in modulation of serotonergic neurons in early development of zebrafish, as the early ontogeny of serotonergic system may be one of the important factors for neuronal growth and brain development. We demonstrated that exogenous administration of E_2_ biphasically affected parameters such as 5-HT-positive areas, relative expression of *tph* isoforms, heart rate and thigmotactic behavior with stimulation and suppression of serotonin system at the low dose and the high dose, respectively, through acting on ER. On the other hand, activities of serotonergic neurons were suppressed by AroB MO-mediated knockdown, suggesting that brain-formed E_2_ in early development stimulates serotonergic neurons, which is in accordance with the results of the low-dose E_2_. Recent study shows that MO-mediated brain aromatase knockdown results in a significant decrease in E_2_ concentration in 48 hpf embryos (57), which supports that our MO experiments reflect the reduction of estrogen production.

Non-monotonic dose responses of hormones and endocrine-disrupting chemicals have been widely documented (58). Estrogen among other hormones is known to exhibit biphasic dose-dependent effects in various physiological processes (59–66). However, only limited information is available in regards to serotonin system. There is one study in fish showing that low dose of E_2_ stimulated monoamine oxidase activity and decreased 5-HT content in hypothalamus in ovariectomized catfish, while the result was opposite for high dose (67). Our study demonstrates that biphasic dose-dependent effects of E_2_ on serotonergic neuron in fish, and shows that the effects or both low and high doses are mediated through ER, indicating physiological relevance. The effect of the low dose of E_2_, stimulating serotonergic neuron, is likely to reflect the role of endogenous E_2_ in embryos, as AroB MO-mediated effects demonstrate that brain-formed estrogen is necessary to maintain activity of serotonergic neuron in embryos. Mechanisms of biphasic responses are complex, but may be in part controlled by downregulation and desensitization of receptors (57, 68). Thus, effects of high doses of E_2_ on serotonergic neuron in this study may be due to downregulation/desensitization of ERs. Sequence analysis of the promoter region of zebrafish *tph* isoforms shows the presence of 1/2 ERE in the upstream of transcription start site in all isoforms, suggesting possible nuclear action of estrogen, though their functional analysis is yet to be reported. In human serotonergic cell line, binding of E_2_ and ERβ has been shown to directly interact with 1/2 ERE of *tph2* promoter to elicit gene expression (39). In addition to the classical action of E_2_ on nuclear receptors membrane ERs plays an important role in brain (69, 70). Interaction between membrane ERs and the metabolic glutamate receptor in the brain provides a rapid and transient E_2_ action (71, 72). Membrane bound G-protein-coupled ER, GPER/GPR30, also known to be involved in modulating rapid non-genomic action of E_2_, plays a role in several brain areas (73). Estrogen action through GPR30 has been suggested in regulation of serotonergic neuron in mammals (74). Further studies are required to elucidate the mechanisms by which estrogen regulates serotonergic neuron in zebrafish.

Attenuation of serotonergic neuron by AroB MO-mediated knockdown clearly demonstrated that brain-formed estrogen is necessary to maintain the serotonin system to control heart rate and anxiety behavior in early development of zebrafish. Validity of AroB knockdown was supported by several lines of evidence. Immunoreactivity to the antiserum specific to AroB was decreased in AroB MO injected embryos. In addition to no significant effects found in the controls including standard MO, inverted AroB MO and AroA MO-injected embryos, AroB MO-mediated effects were rescued by co-injection of AroB mRNA and exposure to E_2_. Off-target effect of MO injection was also examined by knockdown of *p53*, showing that the decreased 5-HT-positive area caused by AroB MO is not through activation of *p53*. The decrease in 5-HT-positive area by AroB MO injection indicates that brain-formed estrogen stimulates 5-HT synthesis, which is in accordance with the stimulatory effect of low-dose E_2_. When the relative expressions of *tph* isoforms were examined in AroB MO injected embryos, only *tph2* expression was significantly decreased by AroB MO, which is well supported by the previous studies showing *tph2* but not *tph1* is expressed in raphe 5-HT neurons (54, 75, 76). Expression of *tph2* in 5-HT neurons in pretectal and hypothalamic complex starts to appear at 60 hpf (76). On the other hand, whereas in the exposure experiments, expressions of all isoforms were affected by E_2_; increased by low dose and decreased by high dose. The results support the previous studies reporting that *tph2* expressed in brain is responsible for 5-HT synthesis in the zebrafish (27, 28, 54). Thus, we provide the evidence that brain-formed estrogen stimulates *tph2* expression to maintain 5-HT content in the serotonin neuron. The effects of E_2_ exposure on *tph* isoforms indicate E_2_ also modulates serotonin biosynthesis in tissues outside the brain. 5-HT has been reported to be produced in various organs including intestine which is the major source of 5-HT in the body and TPH1 is responsible for its synthesis (54, 77). Investigation of estrogen regulation of serotonin production in intestine during development would be a future research interest.

The parameters of physiological functions of serotonin system, heart rate, and thigmotactic behavior were measure to verify the activity of serotonergic neuron. The results were in accordance to the changes in 5-HT levels in the neurons; the increased 5-HT levels are accompanied by the increased heart rate and decreased thigmotactic behavior, while the contrary was true for the decreased 5-HT levels. Serotonin is known to be involved in cardiovascular function, and the effect of central serotonergic neuron is mediated through autonomic nervous system in mammals (18). Our result of the low-dose (0.005 µM) E_2_ which increased heart rate corroborates the effect of MO-mediated AroB knockdown, indicating that nanomolar level of brain-formed estrogen, or even lower level in the tissue, stimulates serotonergic neuron to increase the heart rate. Exposure to quipazine (serotonin agonist), or fluoxetine (selective serotonin reuptake inhibitor, SSRI) completely reversed the decreased heart rate caused by the high dose (0.1 µM) E_2_, or AroB MO injection confirming that heart rate is under the control of serotonin signaling. Taken together with a recent study showing that GPER in the pituitary of zebrafish embryo regulates heart rate through thyroid hormone (78), estrogen in brain centrally regulates heart rate through various mechanisms. On the other hand, cardiac functions are directly regulated by estrogen (79) and aromatase has been detected in the heart tissues such as myocardium in mice (80–82). Therefore, it is possible that AroB MO injection may affect aromatase expression in the heart and locally produced estrogen modulates heart rate. In some teleost fish, both ovarian and brain aromatases are expressed in the heart (83–85), but in ricefield eel only brain aromatase is detected (86), while only ovarian aromatase is present in spotted scat (87). These difference may be due to technical difference as well as differences in species and developmental and physiological status. Our preliminary analysis indicated the expression of ovarian aromatase but not the brain form in adult zebrafish heart (data not shown), suggesting that our result of MO injection is likely to be mediated through knockdown of brain aromatase expressed in the brain not in the heart. However, expression of aromatase in the heart during development needs to be verified.

Thigmotaxis is an evolutionally conserved behavior associated with fear and has been shown to be affected by anxiolytic and anxiogenic compounds (88); thus, it has been used to measure anxiety levels in animals including fish (89–92). Our present study shows the high dose E_2_ (1 µM), which decreased the 5-HT level, significantly increased anxiety (increased time spent in outer zone), and this increase was abolished by addition of 5-HT agonist (Q) or SSRI (FLX), indicating the effect of high dose E_2_ is mediated through serotonin signaling. Similarly, increased anxiety by AroB MO was also abolished by Q or FLX, which supports our hypothesis that brain-formed estrogen modulates serotonergic neuron. Despite our expectation, the low-dose E_2_ (0.005 µM), which increased the 5-HT level, did not cause reduction of anxiety. Therefore, we further examined to see if the low-dose E_2_ exerts anxiolytic effect in the larvae exposed to DEX to induce stress, and indeed, low-dose E_2_ decreased the anxiety level. Thus, our data demonstrate a negative correlation between anxiety behavior and 5-HT level, which is in accordance with previous studies. In mammals, depletion of 5-HT level in rat brain induces anxiety (93) and acute reduction of tryptophan increases the anxiety level in patients of a social anxiety disorder (94). The role of 5-HT in anxiety is also reported in zebrafish (20, 95). Buspirone, partial agonist for 5-HT1A receptor, exerts anxiolytic-like effect in zebrafish (96). The phenotype of zebrafish leopard strain, which is characterized by increased anxiety-like behavior, is rescued by acute treatment with FLX (97). Taken together, we provide the evidence that brain-formed E_2_ has an important role in modulating anxiety through serotonergic transmission.

In contrast to mammalian brain, where aromatase is expressed in both neuron and glia (98, 99), it is well documented that brain aromatase in fish is exclusively expressed in RGCs along the ventricles of forebrain, midbrain, and hindbrain serving as neural progenitors (10, 11, 47). While most RGCs are transformed into astrocytes by the time of adulthood in mammalian brain (100), presence of RGCs persists throughout the lifespan of zebrafish, which is considered to be one of the contributing factors for high capacity of neuronal proliferation (101). On the other hand, serotonin is known to play a role in neurogenesis (102). In adult zebrafish, it has been reported that projection of 5-HT neurons in raphe to ventricular surface of the brain, where highly proliferative cells are found. In addition, expression of 5-HT receptors are localized in ventricular surface in larval and adult zebrafish (27, 103). Thus, it may be possible that RGCs in ventricular surface are innervated by 5-HT neurons in raphe and modulated for neurogenesis. Interestingly, it has been reported that AroB-positive RGCs in PVO area in adult zebrafish has an ability to differentiate into serotonergic neuron (104). Taken together with our present study providing the evidence that brain-formed estrogen is necessary to maintain the levels of 5-HT in neurons in raphe, we can hypothesize that differentiation of AroB-expressing RGCs in serotonin neurons is regulated by serotonin neuron in raphe, whose activity is modulated by estrogen produced by AroB. It has been shown that placenta aromatase activity and expression are stimulated by serotonergic 5-HT2A receptor signaling (105). In goldfish, AroB expression in RGCs *in vitro* is upregulated by dopamine with modulation by E_2_ (106). Nonetheless, estrogen biosynthesis and homeostasis in CNS are regulated and fine-tuned by multiple factors like neurotransmitters and hormones, so that diverse functions of estrogen can be coordinated.

In conclusion, this study demonstrates that estradiol exhibits a biphasic effect on serotonergic neuron, and that brain aromatase, thus brain-formed estrogen plays a significant role in modulating serotonin levels to sustain appropriate development and functions of serotonergic neurons which regulate heart rate and anxiety behavior in zebrafish embryos and larvae. Considering the role of serotonergic neurons in neural development and neurogenesis, it is possible to postulate that one of the mechanisms of brain aromatase and brain-formed estrogen to regulate neurogenesis in teleost brain may be through modulation of serotonergic system, which awaits future investigation.

## Ethics Statement

All experimental procedures and maintenance of fish were conducted in accordance with the Guide for Care and Use of Laboratory Animals published by the US National Institutes of Health.

## Author Contributions

ZU and MK designed the experiments. ZU performed the experiments and analyzed the data. ZU and MK wrote the paper.

## Conflict of Interest Statement

The authors declare that there is no conflict of interest regarding the publication of this article.
